# Impact of COVID-19 on acute angle-closure attack: A retrospective study

**DOI:** 10.1097/MD.0000000000040782

**Published:** 2024-12-06

**Authors:** Shuo Zhang, Feng Mei, Yan Shi, Diya Yang, Ju Zhang, Shuhua Wang, Guoping Qing, Zhigang Fan, Xueting Pei

**Affiliations:** a Beijing Tongren Eye Center Research Ward, Beijing Tongren Hospital, Beijing Institute of Ophthalmology, Beijing Ophthalmology & Visual Sciences Key Laboratory, Capital Medical University, Beijing, China.

**Keywords:** acute angle closure attack, COVID-19 outbreak, incidence, inflammation, retrospective cohort study

## Abstract

To systematically review the characteristics of patients experiencing acute angle closure (AAC) attacks during the COVID-19 outbreak in Beijing. Patients with AAC attacks during the COVID-19 epidemic and those in the same period the following year were recruited. Demographic characteristics, ocular biometry, ocular signs, sequential relationships, and the prognosis of operative management outcome were recorded and compared between the 2 groups. We included 60 eyes of 55 patients with AAC attacks in the COVID-19 group and 34 eyes of 33 patients in the control group. There was a significantly higher incidence of bilateral attacks during the COVID-19 outbreak compared to the control group (9 vs 1, *P* = .043). Additionally, a higher proportion of AAC attacks were observed in the COVID-19 group (*P* = .035). The COVID-19 group had a higher mean peak intraocular pressure (53.42 ± 8.87 mm Hg vs 47.86 ± 11.22 mm Hg; *P* = .007), larger pupil diameter (5.75 ± 1.04 mm vs 4.86 ± 1.26 mm; *P* < .001), more pigmented keratic precipitates (39 vs 8, *P* < .001), and segmental atrophy of the iris (32 vs 10, *P* = .019). Coronavirus infection was simultaneous with or slightly preceded the appearance of AAC attacks. Most patients had an ideal prognosis after comprehensive management. An increased incidence of AAC attacks, more bilateral cases and severe anterior segment inflammation were observed during the COVID-19 outbreak in Beijing. There may be a correlation between the onset of AAC attacks and coronavirus infection, but further research is needed to explore this link.

## 1. Introduction

Glaucoma is a group of ocular diseases that damage the optic nerve and can occur at any age.^[1]^ It is one of the leading causes of blindness in people over 60.^[2]^ The symptoms of glaucoma vary depending on the type and stage of disease. Most types of glaucoma do not present warning signs or obvious symptoms in the early stages. As the disease progresses, patients may experience halos, blind spots, blurred vision, and peripheral visual field defect due to optic nerve impairment. Angle-closure glaucoma (ACG) is a major subtype of glaucoma characterized by elevated intraocular pressure (IOP) due to the closure of the anterior chamber angle. ACG can be classified into primary and secondary forms. Primary angle-closure glaucoma is further divided into acute angle-closure glaucoma (AACG) and chronic angle-closure glaucoma (CACG) subtypes. AACG is a severe form of glaucoma caused by an acute and complete closure of the iridocorneal angle, where the peripheral iris covers the entire trabecular network, leading to an acute angle-closure (AAC) attack.^[3]^ This results in a sharp increase in IOP. Conditions such as a thinner or floppier iris, a thick or anterior lens, and a shallow anterior chamber can cause the iris and cornea to attach, blocking the drainage of the aqueous humor and leading to a sudden increase in IOP.^[4]^ This elevated IOP can cause severe eye pain, red eye and reduced or blurred vision. CACG may not cause immediate symptoms but can develop gradually over time. However, it can also lead to an AAC attack in the later stages of disease progression. Other ophthalmic conditions, such as retinopathy, uveitis and lens subluxation, can also trigger acute episodes of angle-closure, resulting in secondary ACG. AAC is a medical emergency as severe vision damage can occur quickly. Immediate treatment is required to reduce IOP, relieve symptoms, and prevent permanent vision loss.^[5,6]^

The Coronavirus Disease 2019 (COVID-19) is a severe acute respiratory syndrome. The coronavirus has been also isolated from tissues of conjunctiva, iris, and trabecular meshwork of patients by detecting the nucleocapsid protein antigen of virus.^[7]^ Beyond respiratory symptoms, many patients with COVID-19 experience severe eye discomfort and acute vision loss. Statistics reveal that nearly 31.6% of COVID-19 patients present with conjunctivitis.^[8]^ Additionally, some patients suffer from ocular inflammatory diseases such as acute retinal necrosis, choroiditis, and optic neuritis.^[9,10]^ Numerous studies have identified correlations between AACG and COVID-19. The incidence of AACG has significantly increased during the COVID-19 outbreak. Furthermore, patients suffering from both AACG and COVID-19 generally present with significantly higher IOP during treatment.^[11]^ Elderly patients and those receiving flu medications are particularly susceptible to developing AACG when infected with COVID-19.^[12]^ Yousaf et al^[13]^ found that infection with coronavirus has been associated with the syndrome of inappropriate secretion of antidiuretic hormone, which may result in hyponatremia. Low plasma osmotic pressure may lead to fluid migration into the extracellular suprachorioidal space. This choroidal dilation can aggravate the closure of the eye in the case of crowded anterior chambers. Besides, angiotensin-converting enzyme 2 (ACE2) is a key regulator of the renin-angiotensin-aldosterone system (RAAS), which has also been detected in ocular tissues. Exposure to the COVID-19 virus may induce inflammation in aqueous humor circulation through ACE2 receptors present in aqueous humor and ciliary body. Inflammatory reactions can push the lens-iris diaphragm forward, exacerbating congestion in the ciliary ring and causing pupil blockage. However, more detailed clinical information is still needed to deepen our understanding of these correlations.

In this retrospective study, we systematically reviewed detailed clinical information, including pathological characteristics, typical signs, sequential relationships, and prognosis following operative management outcomes in patients with coexisting diseases during this special period. We compared these findings with data from the same period in 2023, with the aim of providing valuable guidance for ophthalmologists to comprehensively understand the coronavirus-induced AACG.

## 2. Materials and methods

### 2.1. Participants

This retrospective study analyzed patients’ data from outpatient, emergency and inpatient medical records, supplemented by completed questionnaires. The COVID-19 group comprised consecutive cases diagnosed with AAC attacks at the outpatient and emergency departments of Beijing Tongren Hospital, affiliated to Capital Medical University, within 1 month after the resumption of normal management following the COVID-19 epidemic (from December 8, 2022 to January 7, 2023). The control group included consecutive AAC cases from the same period in the following year (from December 8, 2023 to January 7, 2024).

This study adhered to the tenets of the Declaration of Helsinki regarding research involving human participants. Written informed consent was obtained from all the participants, and the study received approval from the Ethics Committee of the Beijing Tongren Hospital (approval number: TREC2022-KY109).

### 2.2. Inclusion criteria of COVID-19 group and control group

Patients in the COVID-19 group were those who exhibited symptoms of COVID-19 infection or asymptomatic but tested positive for COVID-19 nucleic acid or antigen during the episode of AAC attack. These patients had previously tested negative for viral nucleic acid. The primary symptoms of COVID-19 include high fever, headache or muscular soreness, cough with sputum, alterations in taste and smell, and dyspnea. In contrast, during the period of AAC attack, all patients in the control group tested negative for COVID-19 nucleic acid or antigen, and did not have any infectious diseases, such as respiratory infections, despite the vast majority of them have had covid-19 previously.

### 2.3. Diagnostic criteria of AAC attack

(1) Goldmann applanation tonometry (GAT) is higher than 35 mm Hg.
(2) Presence of at least one of the following symptoms: eye pain, headache, impaired vision, nausea, or vomiting.
(3) Intraocular signs include: ciliary injection or mixed injection, pressure-induced corneal edema, pigmented keratic precipitates (KP), a mid-dilated pupil, segmental atrophy of iris, and glaucoma spot.
(4) With or without glaucomatous optic neuropathy and visual field impairment.
(5) Absence of previous intraocular surgery.

### 2.4. Data collection

Demographic information and medical history were collected from each patient. The demographic data included gender and age, while the medical history covered the time, symptoms, signs and temperature associated with COVID-19 infection and AAC attacks.

All patients underwent comprehensive ocular examinations, which included assessments of visual acuity, maximum IOP measurement, and slit-lamp examination (evaluating keratic precipitates, iris morphology, pupil diameter, cataract status, etc). Additional evaluations included gonioscopy, fundus photography, Humphrey visual field testing (Carl Zeiss, Jena, Germany), anterior segment optical coherence tomography (AS-OCT; CASIA 2, TOMEY, Nagoya, Japan), IOL-Master 700 biometry measurement (Carl Zeiss, Jena, Germany), and ultrasound biomicroscopy (UBM; MEDA, Tianjin, China) prior to surgery. Measurements of axial length (AL), anterior chamber depth (ACD; accounting for central corneal thickness), and lens thickness (LT) were obtained by IOL-Master biometry. Based on these biometrics, all patients were diagnosed with various subtype of glaucoma, including AACG, CACG and secondary glaucoma.

After admission to the ophthalmic emergency department, patients experiencing an AAC attack may undergo IOP reduction therapy. This can involve a single medication or a combination of medicines, including topical antihypertensive drugs such as myotic, β-blockers, alpha-receptor agonists, carbonic anhydrase inhibitors, or dehydrating agents like mannitol. For patients with clear corneas and pupillary block, laser peripheral iridotomy (LPI) may also be attempted. Patients whose IOP is temporarily controlled below 35 mm Hg with medication will be transferred to the outpatient clinic for further treatment. However, patients whose IOP is not effectively controlled will receive emergency surgical interventions, such as an anterior chamber puncture drainage or peripheral iridectomy. In the outpatient clinic, patients will undergo scheduled operations tailored to the scope of anterior chamber angle closure, the degree of optic nerve damage, and the extent of visual field defects. Surgical procedures may include phacoemulsification cataract extraction (CE), intraocular lens (IOL) implantation, goniosynechialysis (GSL), external filtration, capsular tension ring (CTR) implantation, and pupilloplasty. In details, external filtration surgeries performed include trabeculectomy and Ahmed glaucoma drainage valve (AGV) implantation. All patients were treated surgically by experienced glaucoma specialists, Dr ZGF and Dr XTP.

### 2.5. Statistical analysis

Statistical analysis was performed using SPSS statistical software (Version 27.0; SPSS, Inc., Chicago). Comparisons between the COVID-19 group and control group were conducted using *t* tests for normally distributed variables, χ^2^ tests for qualitative variables, and Mann–Whitney *U* tests for non-normally distributed quantitative variables. All *P* values reported were 2-tailed, and statistical significance was defined as *P* < .05.

## 3. Results

### 3.1. Demographic characteristics of patients with AAC in both groups

A total of 84 cases (94 eyes) were included in the study. Among them, 51 patients (60 eyes) with COVID-19 infection were recruited during COVID-19 pandemic from December 8, 2022, to January 7, 2023 (the COVID-19 group). Additionally, 33 patients (34 eyes) with non-COVID-19 infections were recruited during the subsequent year from December 8, 2023, to January 7, 2024 (the control group). The COVID-19 group comprised 20 males (39.2%) and 31 females (60.8%), whereas the control group included 20 males (60.6%) and 13 females (39.4%). The mean ages (mean ± SD) of the patients were 64.16 ± 8.06 years for the COVID-19 group and 64.09 ± 10.63 years for the control group. Nine patients (17.6%) in the COVID-19 group had AAC with bilateral onset compared to only 1 patient (3.0%) in the control group (*P* = .043). The COVID-19 group included 44 patients (86.3%) with AACG, 2 patients (3.9%) with CACG, and 5 patients (9.8%) with secondary glaucoma. All the patients diagnosed in secondary glaucoma were caused by lens subluxation. In contrast, the control group included 21 patients (63.6%) with AACG, 6 patients (18.2%) with CACG, and 6 patients (18.2%) with secondary glaucoma. In details, among the patients with secondary glaucoma, 2 cases were caused by lens subluxation and another 4 cases were due to neovascular glaucoma (NVG). All the demographic data are presented in Table 1.

**Table 1:** Demographic characteristics of patients with acute angle closure attacks in both groups.

Parameter	COVID-19 group	Control group	*P* value
Cases (eyes)	51 (60)	33 (34)	–
Gender			
Male (%)	20 (39.2)	13 (39.4)	.987*
Female (%)	31 (60.8)	20 (60.6)	
Age (yr)	64.16 ± 8.06	64.09 ± 10.63	.653**
Bilateral onset/total	9/51	1/33	.043*
Diagnosis, cases			.035*
AACG (%)	44 (86.3)	21 (63.6)	
CACG (%)	2 (3.9)	6 (18.2)	
Secondary glaucoma (%)	5 (9.8)	6 (18.2)	

AACG = acute angle-closure glaucoma, CACG = chronic angle-closure glaucoma.

* χ^2^ test.

** Mann–Whitney test.

### 3.2. Ocular biometry of patients with AAC attack in both groups

**Figure 1.**
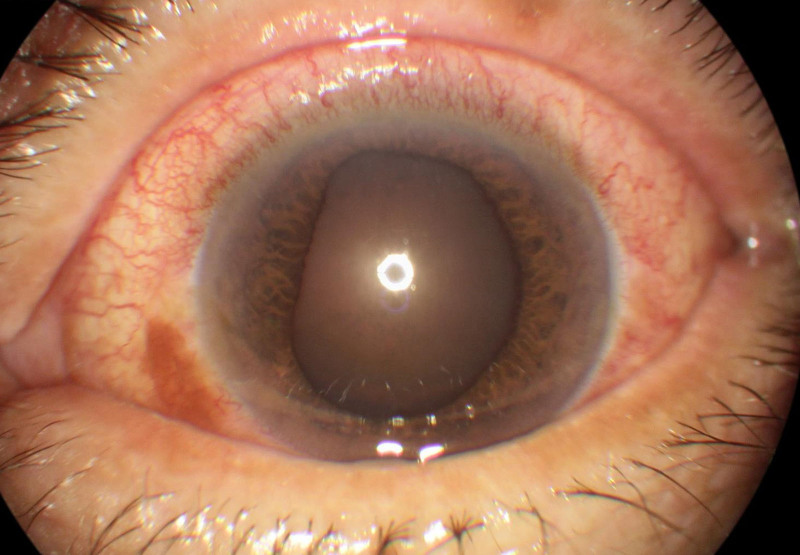
Dilated and fixed pupil of AAC attack eye in the COVID-19 group. AAC = acute angle closure.

All ocular biometry measurements are shown in Table 2. The peak IOP of the COVID-19 group was 53.42 ± 8.87 mm Hg, which is significantly higher than the peak IOP of control group at 47.86 ± 11.22 mm Hg (*P* = .007). The AL were 22.32 ± 0.95 mm and 22.50 ± 0.91 mm in the COVID-19 and control groups, respectively. The ACD were 2.28 ± 0.28 mm and 2.21 ± 0.36 mm, and the LT were 4.99 ± 0.33 mm and 5.00 ± 0.43 mm for the COVID-19 and control groups, respectively. No significant differences in AL, ACD, and LT were observed between the 2 groups. The pupil diameter (PD) in COVID-19 group was 5.75 ± 1.04 mm, which was significantly larger than that of the control group at 4.86 ± 1.26 mm (*P* < .001; Fig. 1). We used lens opacities classification system III (LOCS III), which is the most widely used subjective grading method in clinical practice, to assess the cataract status. There was no significant difference in lens opacity between the 2 groups.

**Table 2:** Ocular biometry of patients with acute angle closure attacks in both groups.

Parameter	COVID-19 group	Control group	*P* value
Peak IOP (mm Hg)	53.42 ± 8.87	47.86 ± 11.22	.007*
AL (mm)	22.32 ± 0.95	22.50 ± 0.91	.472**
ACD (mm)	2.28 ± 0.28	2.21 ± 0.36	.392**
LT (mm)	4.99 ± 0.33	5.00 ± 0.43	.891**
PD (mm)	5.75 ± 1.04	4.86 ± 1.26	<.001*

AL = axial length, ACD = anterior chamber depth, IOP = intraocular pressure, LT = lens thickness, PD = pupil diameter.

* Mann–Whitney test.

** Independent *t* test.

### 3.3. Ocular signs of AAC attack patients

**Figure 2.**
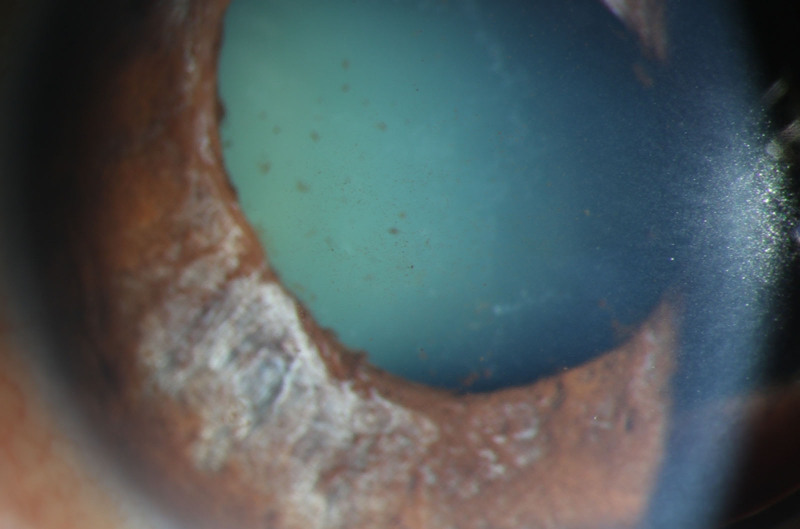
More pigmented keratic precipitates (KP) of AAC attack eye in the COVID-19 group. AAC = acute angle closure, KP = keratic precipitates.

**Figure 3.**
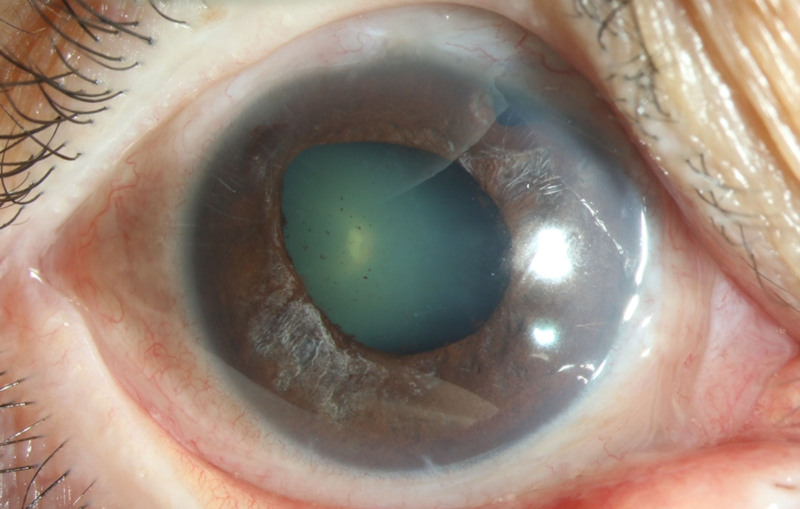
Severe segmental atrophy of iris of AAC attack eye in the COVID-19 group. AAC = acute angle closure.

The vast majority of the patients in both groups exhibited several ocular signs of AAC, including pigmented KP, Tyndall effect, and segmental atrophy of the iris (Table 3). When comparing the 2 groups, the COVID-19 group had significantly more patients with pigmented KP (*P* < .001; Fig. 2) and segmental atrophy of the iris (*P* = .019; Fig. 3).

**Table 3:** Ocular signs of acute angle closure attacks patients.

Positive ocular signs	COVID-19 group	Control group	*P* value
Pigmented KP, eyes	39	8	<.001*
Tyndall effect, eyes	24	9	.158*
Segmental atrophy of iris, eyes	32	10	.019*

KP = keratic precipitates.

* χ^2^ test.

### 3.4. Characteristics of patients with novel COVID-19 infection in the pandemic group

The general information of COVID-19 infection patients with AAC attack is presented in Table 4. COVID-19 infections were concentrated in the first 1 to 2 weeks (6.49 ± 5.78 days) after the outbreak. There were 31 cases (60.8%) in the first week, 14 cases (27.5%) in the second week, 4 cases (7.8%) in the third week, and 2 cases (3.9%) in the fourth week. Among all infections, only 4 cases were asymptomatic, while the remaining 47 cases exhibited various symptoms, including high fever (47 cases, 92.2%), headache or muscular soreness (41 cases, 80.4%), cough and sputum (18 cases, 35.3%), and aberrations of taste and smell (4 cases, 7.8%). The peak temperature of coronavirus infections was 37.82 ± 0.44°C, and it lasted for 1.58 ± 1.06 days. The high fever in patients of bilateral AAC attacks were lasted for longer time than the monocular AAC attacks patients.

**Table 4:** General information of novel COVID-19 infection.

Parameter	COVID-19 group
COVID-19 infect at different weeks, cases (%)	
1st week (%)	31 (60.8)
2nd week (%)	14 (27.5)
3rd week (%)	4 (7.8)
4th week (%)	2 (3.9)
Symptoms, cases (%)	
Asymptomatic	4 (7.8)
High fever	47 (92.2)
Headache or muscular soreness	41 (80.4)
Cough and sputum	18 (35.3)
Aberrations of taste and smell	4 (7.8)
Dyspnea	0 (0.0)
Peak temperature (°C)	37.82 ± 0.44
Duration of high fever (d)	1.58 ± 1.06

### 3.5. The sequential relationships between AAC attack and the onset of novel COVID-19 infection

The AAC attack occurred simultaneously with the coronavirus infections in 25 cases (49.0%), while the remaining AAC attack cases occurred later than the coronavirus infection. Among these cases, AAC occurred <1 day after the coronavirus infection in 13 cases (25.5%), 2 days in 8 cases (15.7%), and more than 3 days in 5 cases (9.8%; Table 5).

**Table 5:** The sequential relationships between acute angle closure attacks and the onset of novel COVID-19 infections.

	COVID-19 group
Simultaneous, cases (%)	25 (49.0%)
≤1 d later than COVID-19 infection, cases (%)	13 (25.5%)
2 d later than COVID-19 infection, cases (%)	8 (15.7%)
≥3 d later than COVID-19 infection, cases (%)	5 (9.8%)

### 3.6. Treatments of AAC attack patients

All cases initially received emergency treatment according to the peak IOP including topical eye drops, and/or systemic drugs, such as mannitol intravenous. If the IOPs were uncontrolled, they underwent LPI or anti-glaucoma surgeries sequentially to reduce IOP. All the treatments were followed European Glaucoma Society Terminology and Guidelines for Glaucoma, 5th Edition,^[14]^ and Chinese Glaucoma Guidelines (2020).^[15]^ However, only 7 eyes in the COVID-19 group and 3 eyes in the control group achieved temporary IOP control below 35 mm Hg through these methods. Additionally, 6 eyes in the COVID-19 group and 1 eye in the control group required emergency surgery due to extremely high, uncontrollable IOP. In the COVID-19 group, 53 eyes underwent scheduled surgeries, compared to 31 eyes in the control group. The surgical procedures included CE, combine IOL implantation, GSL, external filtration, CTR implantation, and pupilloplasty. There was no significant difference in the types of surgeries performed between the 2 groups, except for pupilloplasty. A significantly higher number of eyes in the COVID-19 groups underwent pupilloplasty compared to the control group (*P* < .001). Postoperatively, the mean IOP was normal and stable in the both groups. There was no significant difference in the mean IOP between the 2 groups at different postoperative time point (Table 6).

**Table 6:** Treatments of acute angle closure attacks patients.

Treatment and therapeutic effect, eyes	COVID-19 group	Control group	*P* value
Drugs or LPI (%)	7 (11.7)	3 (8.8)	.635*
Emergency operation (%)	6 (10.0)	1 (2.9)	.199*
Scheduled operation (%)	53	31	
CE combine IOL implantation	41 (77.4)	22 (71.0)	.586*
Combine GSL (%)	37 (69.8)	20 (64.5)	.664*
Combine external filtration (%)	7 (13.2)	8 (25.8)	.149*
Combine CTR implantation (%)	5 (8.3)	6 (19.4)	.196*
Combined pupilloplasty (%)	20 (37.7)	4 (12.9)	<.001*
1 d postoperative IOP (mm Hg)	17.7 ± 2.7	18.2 ± 2.3	.231**
1 mo postoperative IOP (mm Hg)	17.9 ± 1.9	18.1 ± 2.1	.434***
3 mo postoperative IOP (mm Hg)	18.3 ± 2.1	17.8 ± 1.8	.134***

CE = phacoemulsification cataract extraction, CTR = capsular tension ring, GSL = goniosynechialysis, IOL = intraocular lens implantation, IOP = intraocular pressure, LPI = laser peripheral iridotomy.

* χ^2^ test.

** Independent *t* test.

*** Mann–Whitney test.

## 4. Discussion

The surge in AACG cases during the COVID-19 pandemic has been noted in various studies. We enrolled 60 cases of AACG during the COVID-19 outbreak in Beijing and investigated the specific clinical characteristics. In our cohort, nearly half of patients developed AACG simultaneously with COVID-19 infection, particularly among female and older patients. In the COVID-19 group, the prodromal signs of AACG included headache, blurred vision, and fever, which are similar to those of COVID-19 infection and can be easily overlooked in the early stage.

The incidence of bilateral attacks was significantly higher in the COVID-19 group compared to the control group, which aligns with previous reports. The occurrence of bilateral AAC attacks generally increased during the COVID-19 pandemic, suggesting that coronavirus-related systemic inflammation might induce AAC attacks.^[12,16]^ Additionally, the patients in COVID-19 with extremely uncontrolled IOP and more severe anterior segment inflammation, which include more obvious Tyndall effect in anterior chamber, more pigmented KP, and larger pupil diameter. In patients who had both COVID-19 infection and an AAC attack, we found that the coronavirus infection occurred simultaneously with or slightly earlier than the onset of the AAC attack. These sequential relationships partially suggest potential causal links between the 2 conditions. Hyponatremia is the most common complication of COVID-19 infection. Fever, nausea, and vomiting can result in the deficiency of water and electrolytes. Replenishing water after a fever may further lead to dilutional hyponatremia. Additionally, the use of diuretic medications to treat pneumonia can exacerbate electrolyte disturbances.^[17]^ Özmen et al reported a series of cases of AAC accompanying with severe COVID-19 infection, suggesting that hyponatremia, caused by pneumonia, triggered the progression of AAC. Mechanically, hyponatremia created an osmotic pressure difference between the aqueous humor and blood serum, leading to angle-closure and increased IOP.^[18]^ In contrast to their findings, most patients in our study presented with relatively mild symptoms of COVID-19 infection. Hyponatremia or electrolyte disorders were less frequent in our groups. Moreover, in some cases, the acute onset of angle-closure and COVID-19 infection occurred almost simultaneously. Therefore, we propose that the direct invasion of ocular tissue by the coronavirus may also contribute to the development of AAC.

Interestingly, early prone position ventilation has been applied to treat severe COVID-19 pneumonia to improve oxygenation and reduce mortality. The prone position can lead to a forward displacement of the lens-iris diaphragm, which may partially block the anterior chamber angle. Consequently, patients with shallow anterior chambers are more likely to develop AACG when placed in a prone position for the management of severe COVID-19 pneumonia.^[19–21]^

There was no difference in ocular structure between the 2 groups, but patients in the COVID-19 group presented with more severe ocular pathological conditions. The average IOP in patients with coronavirus infection was significantly higher than that of the control group. Moreover, a considerable portion of patients in the COVID-19 group exhibited a complete loss of iris elasticity and marked pupil dilation. Distinct pathological features of anterior segment inflammation, such as segmental atrophy iris, Tyndall effect in anterior chamber and pigmented KP, were observed in the COVID-19 group. Among them, more than 60% of patients had a Tyndall effect more than Grade 2. ACE2 is a key regulator of the RAAS, which plays crucial roles in maintaining the physiological and pathophysiological balance of the body. ACE2 also serves as a functional receptor on cell surfaces through which the COVID-19 virus enters host cells. ACE2 is highly expressed in the lung, heart, kidney, and plasma, contributing to multiple organ injuries during the COVID-19 outbreak.^[22]^ The involvement of ACE2 and RAAS has also been detected in ocular tissues, including the cornea, conjunctiva, retina, and aqueous humor.^[23–25]^ Notably, ACE2 receptors expression is significantly higher in glaucomatous eyes compared to non-glaucomatous eyes.^[26]^ Exposure to the COVID-19 virus may induce inflammation in aqueous humor circulation through ACE2 receptors present in aqueous humor and ciliary body. In patients with a relatively shallow anterior chamber or narrow angle, inflammatory reactions can push the lens-iris diaphragm forward, exacerbating congestion in the ciliary ring and causing pupil blockage. Meanwhile, iris edema and adhesion further aggravate angle narrowing or closure. In several cases of acute IOP increase following COVID-19 infection, acute depigmentation of the iris and positive iris transillumination were observed.^[27–29]^ The anterior chamber showed signs of uveitis and distinct pigmentation.^[30]^

All patients in our cohort received medication, LPI, or surgical treatment based on their IOP and the specific types of glaucoma they had. Most patients achieved an ideal prognosis following comprehensive management. However, in the COVID-19 group, 6 eyes required emergency operations due to the uncontrollable IOP and inflammation. To be specific, 2 of them underwent anterior chamber puncture drainage, while another 4 patients underwent peripheral iridectomy. Furthermore, most of the remaining eyes in the COVID-19 group required scheduled surgeries since conservative treatments could not completely alleviate IOP and nerve dysfunction. Due to the high number of elderly patients in both groups, nearly 80% of the eyes underwent CE combined with IOL implantation. This procedure aimed to thoroughly address pupillary block, enhance anterior segment configuration, and simultaneously restore visual function by removing the clouded lens. Additionally, individualized treatment strategies were essential based on CE combined with IOL implantation. For eyes with severe adhesion of anterior chamber angle, GSL was critical to fully open the angle and restore the aqueous drainage channel. In cases where there was an extensive closure of the chamber angle, external filtration surgery was selected to reconstruct the aqueous drainage system. For eyes with secondary glaucoma due to lens subluxation, phacoemulsification combined with CTR and IOL implantation is recommended. CTR implantation was employed to provide centrifugal force between the remaining suspensory ligaments, redistribute the existing suspensory ligaments to the whole circle of suspensory ligaments, and maintain the expansion of the capsule. It also helps to stabilize the intraocular lens position, and to reopen the angle of the chamber and deepen the anterior chamber. So as to achieve the purpose of treating ACG and to control the IOP.

Additionally, persistent fever and elevated IOP led to severe iris ischemia and damage to the pupil sphincter, resulting in significant pupil dilation. Approximately 40% of eyes in the COVID-19 cohort underwent pupilloplasty, a rate significantly higher than that of the control cohort. In the majority of patients in the COVID-19 group, IOP was significantly reduced to normal levels 1 day postsurgery and remained stable throughout long-term follow-up.

This study has several limitations that warrant consideration. Firstly, there are potential biases inherent in retrospective study. Our study is a retrospective study conducted at a single center, so there is potential selection bias. Because several cases that were not enrolled due to special reasons, such as cases that were far from the study hospital, or cases that were inconvenient to visit during coronavirus infection. It is limiting its ability to generalize the findings to other regions in China. A large-scale, multicenter study is necessary to comprehensively compare the characteristics of AAC in patients with and without coronavirus infections. Besides, there may be information bias, including incomplete medical records or recall bias. While, we used electronic medical records in our department, which helps us reduce relative bias. Also, enrolling patients were questioned repeatedly to ensure the authenticity of the information to reduce recall bias. Secondly, to strengthen the correlation between COVID-19 and AAC, it would be beneficial to conduct further analyses, such as examining the relationship between peak fever temperature and peak intraocular pressure. Thirdly, we compared the clinical presentations between the 2 groups, which showed that there were more bilateral attacks, extremely uncontrolled IOP, and more severe anterior chamber inflammations in the COVID-19 group. However, we only followed up 3 months postoperatively, and it is necessary to conduct long-term observation in the future to explore the differences in progression of AACG between the groups. Additionally, recent evidence has highlighted potential biomarkers expressed in aqueous humor that could elucidate the critical role of immune responses in the onset of COVID-19-related acute glaucoma.^[31]^ Since out study only included clinical data, further research into molecular mechanisms is essential to better understand the specific pathogenesis linking coronavirus infection and AAC. For instance, we can do basic scientific research by monitoring viruses and inflammatory factors in aqueous humor, or by conducting immunohistology studies with trabecular network tissue.

Although COVID-19 is an extreme public health emergency, complications such as the occurrence of COVID-19-related glaucoma deserve special attention for clinicians. In this study, we present a summary of glaucoma cases during the COVID-19 pandemic. We systematically reviewed the epidemiological characteristics, potential pathophysiology mechanisms, and therapeutic strategies in patients with AAC in the context of COVID-19 infection. This study aids ophthalmologists in better understanding COVID-19-related glaucoma and underscores the importance of effective diagnosis and interventions for this condition. At the moment, COVID-19 infection is not a major global public health concern, and many people may forget that this coronavirus exists. But COVID-19 will remain a recurring disease that health care must manage it. Therefore, for some patients with AAC attack who do not respond well to anti-glaucoma treatment, or sudden bilateral red eyes similar to conjunctivitis, it should be alert to the possibility of COVID-19 infection. Because the IOP of COVID-19 infection-associated AAC is difficult to control, timely diagnosis and appropriate treatment can limit the rate of blindness caused by this disease.

## 5. Conclusions

Although COVID-19 is an extreme public health emergency, complications such as the occurrence of COVID-19-related glaucoma deserve special attention for clinicians. In this study, we present a summary of glaucoma cases during the COVID-19 pandemic. We systematically reviewed the epidemiological characteristics, potential pathophysiology mechanisms, and therapeutic strategies in patients with AAC in the context of COVID-19 infection. This study aids ophthalmologists in better understanding COVID-19-related glaucoma and underscores the importance of effective diagnosis and interventions for this condition. It is necessary to identify of COVID-19 infection-associated AAC with unusual severe anterior segment inflammations as early as possible, and ensure timely and effectively treatment. For AAC attack patients with extremely uncontrolled IOP, suitable indications and surgical timing could greatly rehabilitate vision and limit the rate of blindness. Although COVID-19 infection is not a major global public health concern, it is still a recurring disease. For patients with the coronavirus infection, if they have severe non-respiratory symptoms such as headache and vomiting, it is important to be alert to the presence of AAC attack. Meanwhile, health education should be taken to prevent severe loss of visual function in patients with AAC attack.

## Acknowledgments

This project was supported by National Natural Science Foundation of China (Grant number: 82171050; China) and Beijing Municipal Science & Technology Commission (No. Z221100007422057).

## Author contributions

**Data curation:** Shuo Zhang, Feng Mei, Yan Shi, Diya Yang, Ju Zhang, Shuhua Wang.

**Formal analysis:** Shuo Zhang.

**Funding acquisition:** Zhigang Fan.

**Investigation:** Shuo Zhang, Feng Mei, Yan Shi, Diya Yang, Ju Zhang, Shuhua Wang, Guoping Qing.

**Methodology:** Shuo Zhang, Feng Mei, Yan Shi, Diya Yang, Ju Zhang, Shuhua Wang, Guoping Qing, Zhigang Fan, Xueting Pei.

**Project administration:** Xueting Pei.

**Software:** Shuo Zhang.

**Supervision:** Zhigang Fan, Xueting Pei.

**Writing – original draft:** Shuo Zhang.

**Writing – review & editing:** Guoping Qing, Zhigang Fan, Xueting Pei.
